# Drug Overdose Patterns Among Emergency Department Patients at an Academic Hospital in Jeddah

**DOI:** 10.7759/cureus.33584

**Published:** 2023-01-10

**Authors:** Waddaa Reda, Nizar A Bakhashwain, Saad A Albehiri, Hamed A Alghamdi, Ammar A Jumah, Waleed K Eibani

**Affiliations:** 1 Emergency Medicine, King Abdulaziz University, Jeddah, SAU; 2 Medicine, King Abdulaziz University Hospital, Jeddah, SAU; 3 Emergency Medicine, King Abdulaziz University Hospital, Jeddah, SAU

**Keywords:** overdose prevention, drug intoxication, drug overdose and poisoning, emergency deparment, drug overdose

## Abstract

Background

Drug overdose is a significant healthcare issue and remains a common phenomenon in the emergency department (ED). The incidents have increased over the last few years worldwide. There are a few studies about drug overdose in Saudi Arabia in general and Jeddah city specifically. We aimed to describe the pattern of drug overdoses in the emergency department at an academic hospital in Jeddah between 2015-2022.

Methodology

A retrospective record review study was done in 2022 at an academic hospital in Jeddah between 2015-2021, where charts were reviewed for all reported patients presenting to the ED with drug overdose, including all ages and both genders. A careful review of their medical records, data collection, and processing was done using Google Forms (Google, Mountain View, California) and Microsoft Excel (Microsoft, Redmond, Washington), respectively. Statistical analysis was performed using Statistical Package for Social Sciences (SPSS) version 26 software (IBM Inc. Armonk, New York).

Results

Seventy-eight patients were identified, meeting the criteria from the medical records. Most of the patients were children under 12 years of age. Most patients were clinically stable when they arrived at the emergency department. Gastrointestinal symptoms were the most common clinical presentations, followed by drowsiness, while some patients were non-symptomatic. Analgesics and nonsteroidal were the most common causes of drug overdose.

Conclusion

We concluded from this limited study that the most commonly used causative agent in drug overdoses was nonsteroidal and analgesics. Moreover, children younger than 12 years of age constituted the majority of drug overdose patients, and accidental overdose represented the majority of cases. Therefore, it is important to increase public awareness of proper child supervision and keep drugs out of children's reach. More research using larger and more representative data is needed to identify patterns of drug overdose in the community.

## Introduction

Drug overdose is a worldwide health issue and remains a common phenomenon in the emergency department (ED). The incidents have increased over the last few years, adding a burden on morbidity and mortality worldwide, leading to an increase in the load on healthcare organizations worldwide despite being a preventable cause of death. Therefore, it has recently gained global attention [1, 2]. A study conducted in the United States stated that there were 67,367 drug overdose deaths in 2018 [3]. The patients usually suffer from central nervous system and gastrointestinal tract symptoms [4]. As a result, emergency doctors are the first-line responders in such cases. They are constantly facing these cases, which explains the need to assess the magnitude of the problem and outlines the pattern of a drug overdose.

A recent paper from The United States involving 29 states from 2018 to 2019 reveals an increase in the rates of overdose involving amphetamine, cocaine, and opioids, with a decline in benzodiazepine-related overdose. Furthermore, methamphetamine was the most commonly reported drug overdose in 2019 [5]. In Saudi Arabia, drug overdose has been accountable for many intensive care unit (ICU) admissions and ED visits [6]. Recent research conducted in 2021 involving 852 intoxicated patients in Najran, Saudi Arabia, revealed that most of the overdose cases were accidental (64.6%) [7]. Another study in the Western Region of Saudi Arabia found that most cases were in the summertime; moreover, analgesic and non-steroidal anti-inflammatory drugs (NSAIDs) were the most common drugs reported [4].

We believe that the prevalence of drug overdose has increased in the past years, based on our observation, and it will continue to rise among our population, according to multiple research studies regarding drug overdose in Saudi Arabia [4, 6, 7]. In addition, there is quite a lack of updated studies about drug overdose in Saudi Arabia in general and Jeddah City specifically. Over the last several years. Therefore, we conducted this retrospective study to estimate the severity of the problem and characterize the pattern of drug overdoses in the emergency department at our academic hospital in Jeddah between 2015-2022.

## Materials and methods

Study design and setting

A retrospective chart review study was done in 2022 at King Abdulaziz University Hospital in Jeddah on data from 2015 to 2021. The hospital is an academic center with 40+ ED beds. The total annual ED visits are approximately 75,000, and the admission rate from the ED is around 23%. The hospital serves the entire community of Jeddah and provides free healthcare to the general population.

Sample size and sampling procedure

We pulled data from the hospital's electronic medical records using the International Classification of Diseases (ICD) 10 coding system. We included all charts of patients who met the criteria of having a diagnosis of drug overdose and who presented to the ED during the research period. The patients included were of all ages and of both genders. The data was collected using Google Forms (Google, Mountain View, California), and we used SPSS version 26 (IBM Inc., Armonk, New York) for data analysis.

Data collection instrument

Data collected from the electronic medical records (EMR) in the research period included age, gender, nationality, initial clinical presentation, vital signs, name of the overdosed drug, prior history of overdose, treatment given, patient referral, history of psychiatric illness, and the presence of intention (suicidal or accidental).

Analysis

Data was analyzed using the SPSS program version 26. The qualitative data was presented as numbers and percentages, and the quantitative data was reported as mean and standard deviation.

Research ethics

This study has been approved by the Institutional Review Board (IRB) of the center at which the research was conducted.

## Results

A total of 78 cases of drug overdoses were admitted to the emergency department between January 2015 and December 2021. Most of the patients were children under 12 years old (n=56, 71.79%), with mean age (8.846 ± 12.3836), and just over half of the patients were males (n=40, 51.28%). Regarding the patients' nationalities, the majority of them were Saudis (n=52, 66.67%). The annual incidence rate varied from 2015 through 2021, and there were relatively lower cases in 2015 and 2020 compared to the other years. Most patients were admitted to the hospital in the period from April to September (Table 1).

**Table 1:** Demographic data

Variable	Number of patients (%) n= 78
Age	
Under 12	56 (71.79)
35-12	18 (23.08)
Above 35	4 (5.13)
Gender	
Male	40 (51.28)
Female	38 (48.72)
Nationality	
Saudi	52 (66.67)
Non-Saudi	26 (33.33)
Time of occurrence	
Jan-Mar	13 (16.67)
Apr-Jun	25 (32.05)
July-Sep	25 (32.05)
Oct-Dec	15 (19.23)
Occurrence per year	
2015	5 (6.41)
2016	9 (11.54)
2017	17 (21.79)
2018	12 (15.38)
2019	9 (11.54)
2020	6 (7.69)
2021	20 (25.64)

Most patients came to the hospital without a previous history of drug overdose (n=75, 96.15%) and psychiatric illness (n=73, 93.59%). In terms of the cause of a drug overdose, it was established that accidental overdose represented the majority of cases (n=59, 75.64%). In contrast, cases where the drug was taken for suicidal purposes were less common (n=10, 12.82%).

Most patients were clinically stable when they arrived at the emergency department (n=68, 87.18%). However, a variety of symptoms were reported by the patients, including GI symptoms (n=28, 48.71%), while central nervous system (CNS) symptoms measured as (n=27, 34.62%). Other symptoms, including agitation, cough, fever, and tremors were (n=6, 7.69%) and some patients were asymptomatic (n=4, 5.13%) (Table 2).

**Table 2:** Clinical characteristics and presentation

Variable	Number of patients (%) n= 78
Previous history of drug overdose	
Yes	3 (3.85)
No	75 (96.15)
Psychiatric illness	
Yes	5 (6.41)
No	73 (93.59)
Manner of drug overdose	
Suicidal	10 (12.82)
Accidental	59 (75.64)
Other	9 (11.54)
Vital signs	
Stable	68 (87.18)
Unstable	10 (12.82)
Clinical presentation	
Nausea or vomiting	24 (30.77)
Abdominal pain	10 (12.82)
Diarrhea	2 (2.56)
Jaundice	2 (2.56)
Drowsiness	22 (28.21)
Seizure	3 (3.85)
Headache	2 (2.56)
Dyspnea	1 (1.28)
Epistaxis	1 (1.28)
Spontaneous erections with discomfort	1 (1.28)
Other (agitation, cough, fever, tremor.)	6 (7.69)
Asymptomatic	4 (5.13)

Drug overdoses were established to be caused by anti-convulsant, cardiac, and anti-diabetic drugs in 12 (15.38%), four (5.13%), and five (6.41%) of all patients, respectively. Additionally, it was found that some drugs were less frequently to be responsible for drug overdoses, such as anti-hypertensives (n=3, 3.85%), antibiotics (n=1, 1.28%), synthetic hormones (n=5, 6.41%), anti-depressants (n=1, 1.28%), anti-psychotics (n=1, 1.28%), anti-asthmatics (n=1, 1.28%). In four cases (5.13%), the cause of drug overdose was co-ingestion drugs, including paracetamol, opioid, solpadeine, amoxicillin, cefaclor, feroglobin, and cefuroxime. Moreover, 17 patients presented with overdose of other drugs (21.79%), and one patient was noted as having overdosed on an unidentified substance.

The management approaches for many patients involved more than one treatment option, including observation (n=21, 26.92%), supportive treatment (n= 10, 12.82%), antidote (n=10, 12.82%), and multi-interventional approach (n=37, 47.44%). In addition, most patients were referred to the appropriate specialty to complete the management (n=51, 65.38%) (Table 3).

**Table 3:** Drug used and patient management

Variable	Number of patients (%) n= 78
Drug used	
Analgesics nonsteroidal	23 (29.49)
Anti-convulsant	12 (15.38)
Cardiac drugs	4 (5.13)
Anti-diabetics	5 (6.41)
Anti-hypertensive	3 (3.85)
Anti-biotics	1 (1.28)
Synthetic hormones	5 (6.41)
Anti-depressants	1 (1.28)
Anti-psychotics	1 (1.28)
Anti-asthmatics	1 (1.28)
Co ingestion	4 (5.13)
Other (vitamins, supplements, cough syrup)	17 (21.79)
Unknown	1 (1.28)
Intervention	
Observation	21 (26.92)
Supportive treatment	10 (12.82)
Antidote	10 (12.82)
Multi-interventional approach	37 (47.44)
Referral to another department	
Yes	51 (65.38)
No	27 (34.62)

## Discussion

This study aimed to assess the pattern of drug overdoses between 2015-2021 in the emergency department at an academic hospital in Jeddah.

Self-induced drug overdose is a growing issue among young Saudis. A total of 78 cases of drug overdose between 2015-2021 have been reported in our study. The rapid development of the Kingdom of Saudi Arabia's health system has made it simpler to obtain pharmaceuticals and increased the likelihood of incorrect use [8].

Most of the patients were children under 12 years old (n=56, 71.79%), unlike research conducted in Ireland, which has a minority of cases for the age group under 15 years old [9]. We believe that the inconsistent result with the study from Ireland could be due to the fact that their study focused on intentional drug overdose while our study includes both intentional and accidental forms of drug overdose. A recent study conducted in Makkah in 2022 indicated that the most affected age category with drug poisoning was over 15 years old (67%), with it being more accurate and relatable to our results by including intentional and unintentional drug intoxication [10]. As a result, it is crucial to raise public awareness about proper child supervision and keep drugs out of children's reach.

Another finding in our study shows that the most common drug overdose was for analgesic and non-steroidal anti-inflammatory drugs 23 (29.49%), similar to another study conducted in Asir Region in Saudi Arabia in 1995 [11]. We assume that the reason for the similarity is due to the same demographic and cultural study sample. We suggest that the authority increases awareness and promotes the proper usage of over-the-counter medications and drug overdose to decrease the possibility of drug intoxication.

As the results of our show that there were relatively lower cases in the year of 2015 and the year of 2020 compared to the other years. At the same time, most of the cases happened in 2021, which is opposite to another study conducted in the United state that revealed a reduction in the number of reported cases in 2021 [12]. In addition, our study shows that most of the cases were between the period from April to September. We propose this as a consequence of the summer season since it has the school vacation season while some children remain indoors with few outside activities. Consistent with our findings, the majority of overdose cases often occur during the summertime [13, 14].

A variety of symptoms were reported by the patients; most of the patients were complaining of GI symptoms followed by CNS symptoms, in contrast with a study done in Najran, Saudi Arabia, which reported that the most common symptoms were CNS followed by GI symptoms. Also, we established that accidental overdose represented the majority of cases (n=59, 75.64%), while the cases where the drug was taken for suicidal purposes were less common (n=10, 12.82%). Therefore, it is important to increase public awareness of proper child supervision and keep drugs out of children's reach. Although this data was reported from a single academic institute with a small sample size, it could be beneficial for forming hypotheses for further studies.

Limitation

In seven cases, we could not determine if the reason for the overdose was accidental or intentional, which amounts to about 10% of the cases in our study sample. Furthermore, based on our direct observation, we believe that the number of actual cases of drug overdose is higher than what we retrieved from the database of the hospital. This can either be from underreporting, misreporting, misdiagnosis of the presenting patients, or difficulties with data extraction from our institutional EMR system using the ICD 10 coding system. Strategies to improve reporting should be developed to aid in obtaining drug overdose data in the future for public health and research purposes.

Our study was conducted in a single academic institution. Although the hospital provides care to the general public, it doesn't reflect the entire population of the researched geographic area as many patients would choose to go to a Ministry of Healthy hospital or the private sector, which our institution doesn't fall under.

Finally, testing for toxicological substances is done by sending the patient's blood or urine samples to a regional laboratory. In many cases, the results of these tests were not recorded.

## Conclusions

According to the current study, the most commonly used causative agent in drug overdoses were non-steroidal and analgesics, and many patients received management involving more than one treatment option. Moreover, children younger than 12 years old constituted the majority of drug overdose patients, and accidental overdose represented the majority of cases. There is a growing problem of self-induced drug overdose among young Saudis, and the drugs used are those commonly prescribed and available at home. Therefore, it is important to increase public awareness of proper child supervision and keep drugs out of children's reach. Although these data were reported from a single institute and limited area, it could be beneficial for forming hypotheses for further studies. Better reporting systems are needed to improve the accuracy and power of future research.
